# The potential of green ammonia in the de-fossilization of the steel, glass and cement industries

**DOI:** 10.1098/rsta.2023.0270

**Published:** 2024-09-23

**Authors:** Joseph El-Kadi, Krishna V. Kinhal, Luc Liedtke, Juan Luis Pinzón-Ramírez, Collin Smith, Laura Torrente-Murciano

**Affiliations:** ^1^ Department of Chemical Engineering and Biotechnology, University of Cambridge, Philippa Fawcett Drive, Cambridge CB3 0AS, UK

**Keywords:** ammonia, decarbonization, industry

## Abstract

The development of new technologies for the synthesis of green ammonia using exclusively hydrogen from water and nitrogen from air in processes driven exclusively by renewable energy is poised to decarbonize the production of this important molecule for the production of green fertilizers as well as offering a carbon-free vector for the long-term storage of renewable energy. In this article, we explore and quantify the CO_2_ emission reduction potential of green ammonia, evaluating how it can facilitate the decarbonization of other hard-to-abate industrial processes such as steel, glass and cement industries. Green ammonia can be used as a direct replacement of fossil fuels used as energy sources in the different processes. In addition, green ammonia can facilitate the electrification of the processes (so-called Power-to-X) by storing renewable energy in the long term to balance a decarbonized grid against intermittent renewable energy supplies.

This article is part of the discussion meeting issue ‘Green carbon for the chemical industry of the future’.

## 1. Introduction

**Figure 1.**
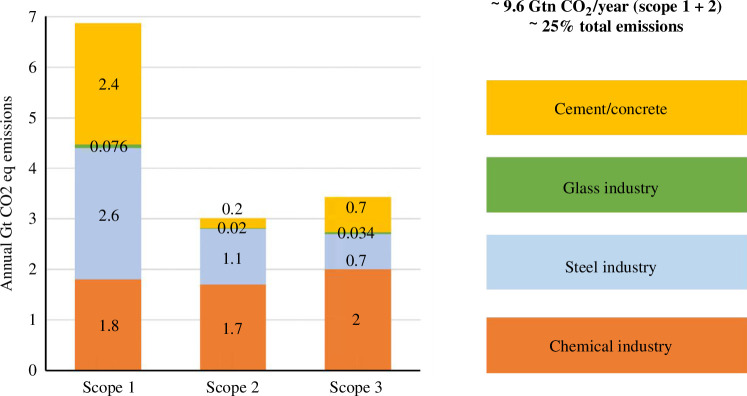
Distribution of Scopes 1, 2 and 3 of CO_2_ equivalent contributions of the cement/concrete, glass, steel and chemical industry (in 2022). Scope 1 emissions are directly emitted by the process as either feedstock or fuel. Scope 2 emissions are associated with electricity used in the process. Scope 3 emissions include indirect upstream and downstream emissions.

The annual global CO_2_ emissions were 36.8 Gt in 2022, corresponding to a 1% increase from the previous year [1]. Out of this value, the chemical industry is responsible for ~5.5 Gt CO_2_-eq (~15%) with a breakdown of ~1.8 Gt CO_2_-eq for Scope 1, ~1.7 Gt CO_2_-eq for Scope 2 and ~2 Gt CO_2_-eq for Scope 3 (figure 1) [2]. Scope 1 emissions are directly emitted by the process for either chemical conversion or energy production, Scope 2 emissions are associated with electricity utilized in the process and Scope 3 emissions are all indirect upstream and downstream emissions, such as those associated with extracting fossil fuels and end-of-life disposal. The distribution of Scopes 1 and 2 contributions depends on whether aspects such as the production of heat and steam are considered within the process boundaries. In general terms, Scopes 1 and 2 emissions are strongly dependent on the fossil feedstock used. For a given energy value, the associated CO_2_ emissions increase as natural gas < oil < coal. A number of technology developments and heat integration strategies over the last decades have facilitated the maximization of the energy efficiency with an associated decrease in emissions. As an example, in the EU27, the chemical industry Scope 1 emissions were ~0.12 Gt CO_2_-eq in 2023, down by 55% since 1990, mainly due to decreased process emissions rather than decreased energy consumption. Equally important, N_2_O emissions were ~1/3 of the total emissions in 1990 but are now minimal due to new legislation and technologies [3].

In addition to the chemical industry, there are other important industries, namely, the cement/concrete, glass and steel industries, which are key for society, but high CO_2_ emitters and difficult to abate. As a whole, the four industries (chemical, cement, glass and steel) are responsible for ~25% of total CO_2_ emissions, with ~9.6 Gt CO_2_ directly (Scopes 1 and 2) emitted annually (figure 1). To put this into perspective, the aviation industry and the shipping industries are each responsible for ~2% of the total CO_2_ emissions [4,5].

Within the chemical industry, the production of ammonia is a particularly energy-intensive process, responsible for ~10% of the CO_2_ emissions of the chemical industry and ~40% of the primary chemical industry, followed by methanol (28%) and high-value chemicals (27%) [6]. Ammonia is mainly used for fertilizers production, particularly as urea, ammonium nitrate and ammonium sulphate. Currently, this process is responsible for ~420 Mt CO_2_ emissions (in 2022) with 1.6 – 1.8 t_CO₂-eq_/t_NH3_ when using natural gas as feedstock and fuel and 3.2 t_CO₂-eq_/t_NH3_ when using coal [7]. It is important to note that China is currently the main ammonia producer with almost all their plants using coal as feedstock and fuel.

In addition to current uses, ammonia is currently considered to be the carbon-free energy carrier of the future due to its high hydrogen content and volumetric energy density. Ammonia is easy to liquefy at mild pressures (> 8 bar) simplifying its storage and transport, with an existing transport infrastructure due to its use as fertilizer. Particularly interesting is its potential as a long-term energy storage medium to balance the seasonal mismatch between energy demand and renewable energy production [8].

In this article, we provide an overview of how the future deployment of green ammonia (i.e. production of ammonia using exclusively nitrogen from air, hydrogen from water and renewable energy to drive the process) has the potential of not only providing green fertilizers but also plays a key role in decreasing the CO_2_ emissions of the hard-to-abate cement, glass and steel industries. In the first part of the article, we present a summary of the current state-of-the-art in the production of green ammonia and the current technological challenges to make it economically feasible. The second part of the paper explores the potential role of green ammonia in the de-fossilization of other industries with a tentative quantification of its potential in decreasing CO_2_ emissions using current assets and technologies. Finally, we highlight how ammonia can enable the direct electrification of processes by storing intermittent renewable energy, while presenting some of the key challenges remaining for the deployment of green ammonia as an energy carrier.

## 2. Recent progress on green ammonia

Grey and brown ammonia are commonly known as ammonia produced using coal and natural gas, respectively, with 70% of ammonia today produced using natural gas and 26% produced using coal (3% from oil and 1% from electricity) [7]. The production process based on natural gas is considered the best available technology for producing ammonia after undergoing optimization for over 100 years, particularly during the global energy crises and expansion of natural gas extraction in the 1960s–1970s [8]. In brown ammonia production, methane (the primary component of natural gas) and steam are converted to hydrogen, carbon dioxide and nitrogen (by partial-combustion in air) through a series of reformers [9]. After carbon dioxide is removed, the remaining hydrogen, nitrogen and impurities (argon and methane) are compressed to the high pressures (100–250 bar) required for the well-known Haber–Bosch process. In this process, the reactor, operating at high temperatures (> 400°C) and high pressures (100–250 bar) achieves only ~20% single-pass conversion to ammonia due to thermodynamic limitations. As a consequence, ammonia is separated from the outlet reactor stream by condensation prior to recycling the unreacted gases and purging a small amount of gas to prevent the build-up of impurities in what is called the Haber–Bosch loop. These two process steps, the fossil fuel reforming for hydrogen production and the Haber–Bosch loop for ammonia production, are highly integrated. Under steady-state conditions, fossil fuel reforming provides the stoichiometric N_2_:H_2_ ratio, and the waste heat generated is used to drive compressors in the Haber–Bosch loop through steam cycles. The combined fuel and feedstock carbon dioxide emissions of brown ammonia production are ~1.5 t_CO₂_/t_NH3_ [8].

**Figure 2.**
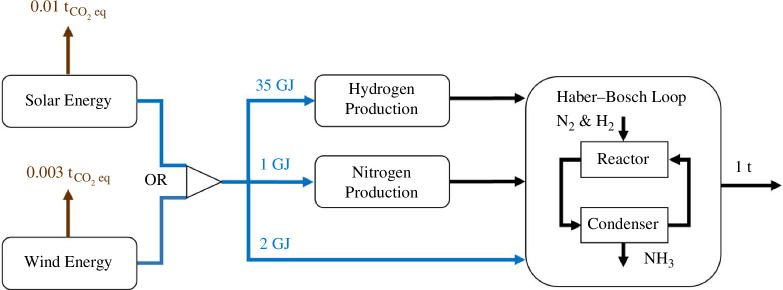
Schematic of green ammonia production processes.

By contrast, green ammonia is produced using hydrogen from water splitting and nitrogen separated from air with the whole process driven by renewable electricity (figure 2) as part of the Power-to-X approach for the electrification of the chemical industry. Green ammonia is estimated to decrease carbon dioxide emissions by 75–90% compared with brown ammonia production, with no direct Scope 1 emissions and only Scope 2 emissions associated with the production of electricity using solar panels and wind turbines, which amount to ~42 g_CO₂_/kWh and ~12 g_CO₂_/kWh, respectively [8,10,11]. Figure 2 illustrates the energy requirements and CO_2_ emissions associated with one ton of ammonia.

The synthesis of green ammonia has been technologically feasible for many years and, indeed, a number of green ammonia plants using a mainly constant energy supply generated by hydropower previously existed in the early twentieth century [12]. Most of the plants shut down around the 1960s when they were replaced by brown ammonia plants based on economic grounds due to the abundance of cheap natural gas. Current environmental pressures and the use of ammonia as an energy vector are reinvigorating the interest for the production of green ammonia, urged by the large cost decrease of renewable electricity from solar and wind experienced over the last decade [13]. Despite this, the main economic bottleneck of green ammonia is the electrolysis of water to produce hydrogen that remains both expensive and energy intensive (with energy efficiencies ~60–70%). More importantly, future green ammonia processes will need to be able to cope with the intermittent renewable solar/wind energy supply. In many cases, the use of intermediate hydrogen buffers and batteries to enable the conventional steady-state operation of the Haber–Bosch loop severely hampers the overall economics [14]. As a result, recent research is focused on not only novel production methods capable of dynamic operation [15,16] but also novel heat integration strategies decoupled from fossil-fuel reforming and enhanced optimization techniques that consider the unique temporal variation of solar and wind energy at specific locations [17,18]. Still, deploying green ammonia production in practice will be capital intensive, leading some to suggest small, modular and distributed ammonia production units or hybridization of existing brown ammonia plants to ease the financial burden of green ammonia production [19].

## 3. Steel industry

Global steel production in 2021 was approximately 1.95 Gt with an average annual growth rate of 2.5–3% over the last 10 years [20]. Assuming a similar growth rate from 2020 to 2050, this trend projects a global steel production of up to 4.60 Gt in 2050. The steel industry is highly polluting with annual total CO_2_-eq emissions of ~3.9 Gt [21], distributed as 67% for Scope 1, 5.7% for Scope 2 and 27.3% for Scope 3 [21,22]. The reason Scope 1 emissions are so high in the steel industry is because the reducing agent used for iron oxide reduction (ironmaking) is typically derived from coal or natural gas feedstocks. In addition, these carbon-based feedstocks are simultaneously used to supply the energy required for the energy-intensive reduction reaction.

Two different processes are currently used for the industrial production of steel: the blast furnace–basic oxygen furnace (BF-BOF) process and the direct reduced iron–electric arc furnace (DRI-EAF) process. Both account for ~80% of global industrial steel production and use iron ore as their main metallic input [21]. A remaining 20–25% of metallic input is provided by scrap steel. In our calculations, we have considered only iron ore as feedstock to provide a fair comparison between the two different process routes regarding the impact on CO_2_ emissions of using green ammonia in place of fossil fuels [21].

### (a) Blast furnace–basic oxygen furnace process

**Figure 3.**
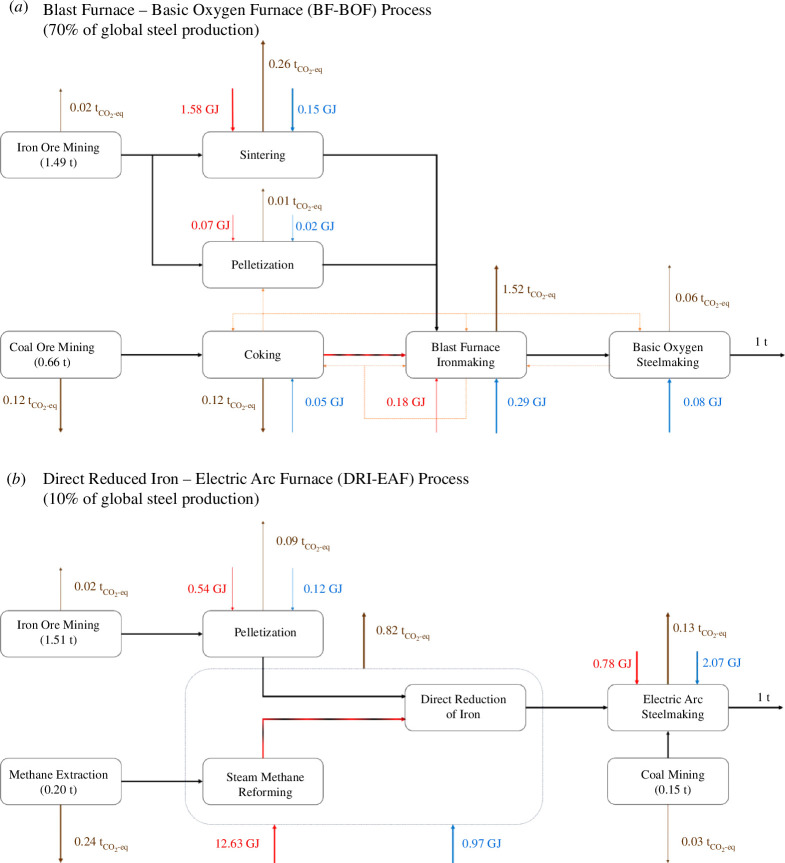
Simplified process diagram for the (*a*) blast furnace–basic oxygen furnace (BF-BOF) and the (*b*) direct reduced iron–electric arc furnace (DRI-EAF) processes. Key process streams are shown in black arrows. Key fuel inputs and electricity inputs are shown in red and blue arrows, respectively. CO_2_ emissions are shown in brown arrows. Heat integration streams are shown in orange arrows. The red and black hatched arrows leaving the coking and steam methane reforming process reflect the fact that coke and syngas act as both reducing agents and energy sources for the ironmaking process. Values are normalized for the production of 1 ton of crude steel.

In this process (figure 3*a*), iron ore is reduced in a blast furnace using coke derived from coal (at > 1200°C in an oxygen-free coking oven) [23]. As mentioned previously, the coal-derived coke has a dual role, acting as both a reducing agent and a heat source for the iron oxide reduction process, which takes place at temperatures between 200 and 2250°C [23]. Coke is needed because it has a higher calorific content than coal, a lower concentration of impurities and is able to better deplete iron ore of oxygen. The resulting liquid metal following blast furnace ironmaking is a brittle, carburized form of iron called pig iron with a carbon content of up to 5% [23]. To improve the mechanical properties of the metal, the pig iron is then exposed to oxygen in a basic oxygen furnace to reduce its carbon content to levels less than 0.4%, thereby yielding liquid steel [23]. This steelmaking process (BF-BOF) is highly integrated with the calorific contents of the coke-off gas, blast furnace gas and basic oxygen furnace gas providing the energy required for the coke ovens and around 90% of the blast furnace energy requirement [23]. Overall, the process leads to an average of 2.2 t_CO₂_/t_steel_ and contributed to 70% of global crude steel production in 2020 [21]. The energy requirement for this process is around 15 GJ/t_steel_, mainly associated with the coke-reducing agent, which supplies over two-thirds of this requirement [21,23].

#### (i) Emissions reduction through the replacement of fossil fuel use with ammonia

**Figure 4.**
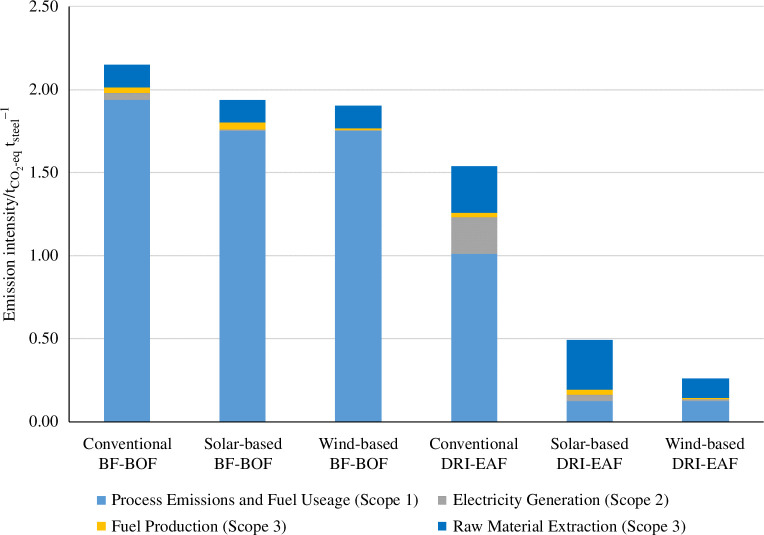
Associated CO_2_ emissions of steel making when using fossil fuels and solar- and wind-derived ammonia.

Without disrupting the heat integration of a BF-BOF plant, ammonia can be used to replace the small amounts of fuel (currently coke and natural gas) that are currently directly used to supply portions of the energy for the sintering, pelletization and blast furnace ironmaking processes. For this, one needs to ensure that ammonia can provide the operating temperatures required for the sintering and pelletization processes (~1450°C) [23], which should be possible considering that the adiabatic flame temperature of ammonia is 1800°C [24]. Assuming technological feasibility, replacing the fossil fuel consumption with green ammonia in this process will lead to a reduction in CO_2_-eq emissions of 8.3 and 9.8% using solar-derived and wind-derived ammonia, respectively, as shown in figure 4.

In addition to this, Scope 2 emissions associated with the production of electricity used in the process (0.59 GJ/t_steel_) [23] could be mitigated using electricity generated by solar or wind energy, which leads to further emissions reductions of 1.6 and 1.8%, respectively. The important role that ammonia has in facilitating this transition to renewable electricity is discussed in §6. Calculations are provided in the electronic supplementary material, S.4.

### (b) Direct reduced iron-based–electric arc furnace

In this process (figure 3*b*), mined iron ore is directly reduced at around 700°C using syngas produced through steam reforming of natural gas (or coal) at 500°C in a highly endothermic process [25]. Both the carbon monoxide and hydrogen in the syngas contribute to the reduction of iron oxide, producing a porous iron product called sponge iron. This sponge iron is then melted in a high-power electric arc furnace to convert the liquid iron into liquid steel. Carbon-containing materials, usually in the form of coal or coke, are also added in this step to adjust the carbon content of the steel. This steelmaking process (DRI-EAF) leads to an average of 1.4t_CO₂_/t_steel_ and accounted for approximately 10% of global crude steel production in 2020 [21]. The energy requirements for this process are 18–30 GJ/t_steel_ [21], of which 12.6 GJ/t_steel_ are required for syngas generation from natural gas [26].

#### (i) Emissions reduction through the replacement of fossil fuel use with ammonia

**Figure 5.**
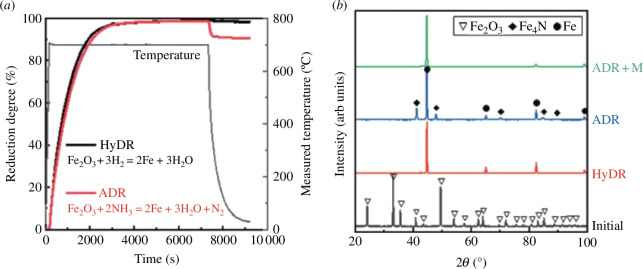
(*a*) Degree of iron pellet reduction when exposed to hydrogen (HyDR = hydrogen direct reduction) and ammonia (ADR = ammonia direct reduction) at 700°C. (*b*) XRD patterns showing the reduction product is iron for HyDR and iron nitride for ADR. The iron nitride phase is dissolved in the electric arc furnace (ADR + M = ammonia direct reduction after melting). Adapted from Ma *et al*. [28].

For this process, ammonia can replace natural gas both as a hydrogen source for the iron reduction and as a fuel. Using ammonia as a reducing agent instead of methane-derived hydrogen is particularly appealing because 50% of the CO_2_-eq emissions from the overall process arise from the steam methane reforming and direct iron reduction stages [27]. Replacing the use of natural gas for iron ore reduction with ammonia has been already demonstrated, during which ammonia decomposes into hydrogen in an autocatalytic process facilitated by the reduced iron acting as a catalyst for ammonia decomposition [28]. The hydrogen released from this decomposition reduces the iron ore in a similar manner to the conventional syngas CO/H_2_ mixture [28]. Indeed, kinetically, the use of ammonia for iron ore reduction is as effective as using H_2_ (see figure 5*a*) but benefits from the *in situ* nitridation of the pure iron (figure 5*b*), which protects it from reoxidation or interaction with ambient moisture during transport [28]. The nitride phase is later dissolved in the electric arc furnace without affecting the quality of the resulting steel [28]. These studies suggest that using H_2_/NH_3_ mixtures would be equally efficient for the direct reduction of iron ore but can help in optimizing the utilization of intermittent renewable energy production. Using solar-derived or wind-derived ammonia as a reducing agent instead of methane-derived syngas leads to a reduction in total CO_2_-eq emissions of ~52 and 64%, respectively. Alternatively, the reduction of iron could be electrified through direct electrochemical reduction of iron oxide to iron via molten salt electrolysis at 800°C with an energy consumption of 14.1 GJ/t_steel_ [29].

Like the BF-BOF process, ammonia can also replace the use of natural gas to supply energy for the electric arc furnace as well as replacing the coal and oil usage in non-integrated pelletization plants (such as those found in DRI-EAF plants) [23]. Doing so leads to reductions in total CO_2_-eq emissions of ~5.5 and 7% for solar-derived and wind-derived ammonia, respectively. In addition, renewable electricity could replace grid electricity, leading to an additional ~12 and 13.5% CO_2_-eq emission reduction using solar-derived and wind-derived electricity, respectively. With all these process modifications, it is possible to reduce the total CO_2_-eq emissions of the DRI-EAF process by 69.5 and 84.5%, respectively, for a solar-powered or wind-powered process using ammonia in place of fossil fuels.

The use of scrap steel as feedstock for the EAF (S-EAF) accounts for 15% of global steel production with a total energy requirement of 2 GJ/t_steel_ and emits 0.3 t_CO₂_/t_steel_ [21]. The process is similar to the DRI-EAF process but uses scrap metal as the iron charge for the electric arc furnace rather than reduced iron. The challenges facing adoption of S-EAF and DRI-EAF are poor availability of scrap or sufficiently pure iron oxide feedstock [21].

For all three processes, a finishing stage is needed where the liquid steel is processed to remove slag and adjust the chemical composition before it is cast into the desired shapes and treated through annealing or galvanization depending on the final application. This step has not been considered in any calculations.

Whilst 70% of global steel production is presently associated with the BF-BOF process, the IEA Sustainable Development Scenario outlines the necessity for a decrease in this contribution to 40% by 2050. In their scenario, the contribution of DRI-EAF-related processes would rise from 10 to 20% alongside S-EAF processes, which rise to 40% [21]. The profound CO_2_-eq emissions reductions achievable with ammonia usage for the DRI-EAF process may encourage further increases in the contribution of DRI-EAF processes to global steel production, especially in countries that are heavily investing in renewable energy sources.

## 4. Glass industry

The global annual production of glass is estimated to be almost 150 Mt [30], with an estimated contribution of approximately 130 Mt of CO_2_-eq/year, with 58% associated with process emissions and fuel usage (Scope 1), 16% associated with electricity generation (Scope 2) and 26% associated with fuel production and raw material extraction (Scope 3). Since glass production can vary significantly depending on the type of glass being produced (i.e. container glass, flat glass, glass fibres or specialty glass), the analysis herein considers a representative average of these types of glass.

**Figure 6.**
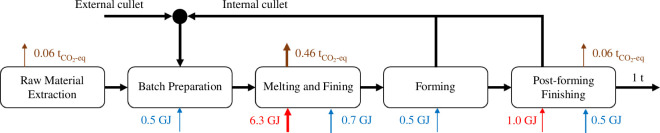
Simplified process diagram of glass production. Key process streams are shown in black arrows. Key fuel inputs and electricity inputs are shown by red and blue arrows, respectively. CO_2_ emissions are shown by brown arrows. Values are normalized for the production of 1 ton of glass.

The standard production of glass can be simplified into four main steps [31], as depicted in figure 6:

- Preparation: where raw materials consisting mainly of silica, limestone and soda ash and cullet material (i.e. ‘recycled’ material both from discarded glass within the plant (internal cullet) and from downstream industries and consumers (external cullet)) go through a number of physical steps to achieve a certain size. The material is normally humidified to avoid dust formation.
- Melting and fining: where the raw and cullet materials are melted at temperatures ranging from 1200 to 1600°C for the formation of oxides (e.g. CaCO_3_ → CaO + CO_2_) and with the objective of achieving a final homogeneous chemical composition. This is the most energy-intensive step of the process. Energy is supplied by burning fossil fuels using gas burners installed above the molten glass with heat transfer dominated by radiation, via electrical currents where electrodes are immersed directly into the melt or a combination of both.
- Forming: where the molten glass is shaped at temperatures between 600 and 1200°C.
- Post-forming and finishing: which includes annealing, tempering, lamination and curation at temperatures between 100 and 600°C. This last step is highly dependent on the type of glass being produced.

The energy consumption and thus CO_2_ emissions associated with glass production have been decreasing over the last 80 years, currently approaching the minimum theoretical energy demand. Such values vary depending on the type of glass, varying from 2.25 GJ/t_glass_ for borosilicate glass to 3.24 GJ/t_glass_ for crystalline glass containing barium (without cullet) [31] due to the differences in their heat of reaction and enthalpy of the glass and the emitted gases.

Using cullet (i.e. recycled) glass is also an efficient way of reducing energy demand and emissions as the recycled material does not have to be chemically reduced during melting. As a general rule, every 10% of cullet glass used as input material reduces reaction and raw material extraction emissions by 10% and combustion and fuel production emissions by 2.5–3% [32], which accounts for a 3.7% reduction of the total CO_2_ emissions for a process run with natural gas. At present, cullet accounts for 10% of flat glass and 50% of container glass inputs in Europe (with lower values at a global scale), but this could be increased to 50 and 80%, respectively [31].

### (a) Emissions reduction through the replacement of fossil fuel use with ammonia

The melting and fining stage is the most energy-intensive one in the glass-making process. The electrification of this step is currently well spread with the use of electric melting furnaces with a considerably higher thermal energy efficiency than combustion furnaces (85 versus 45%, respectively) [31]. The lower efficiency of the combustion furnaces is associated with the latent heat of the exhaust gases at high temperatures. However, electric melting furnaces have a limited capacity (~250 t_glass_/d), and they are not easily adaptable to varying feed compositions and thus cannot be deployed for all types of glass [31]. The potential role of ammonia in the electrification of unit operations to mitigate the intermittent renewable energy supply is discussed in §6.

**Figure 7.**
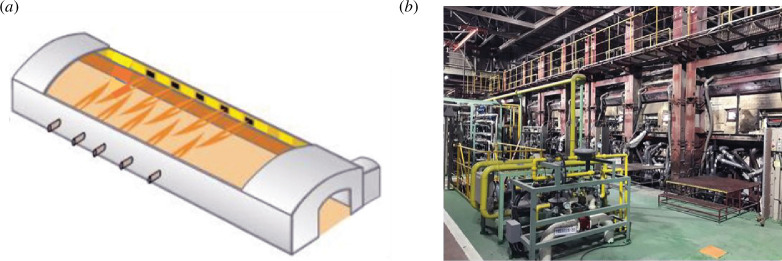
(*a*) Schematic of the combustion furnace fired with ammonia. (*b*) Picture of the pilot demonstration of the glass melting furnace by AGC. Images provided by AGC Inc. [34].

Alternatively, green ammonia can be used directly in combustion furnaces to replace the current use of fossil fuels, as has been recently demonstrated in Japan by AGC [33]. They have demonstrated the use of 100% ammonia in 200 kW burners for glass production [34], where pairs of burners are installed on both sides of the molten glass (figure 7). The company claims to be working towards the scale-up of the ammonia burners to 1 MW [35].

**Figure 8.**
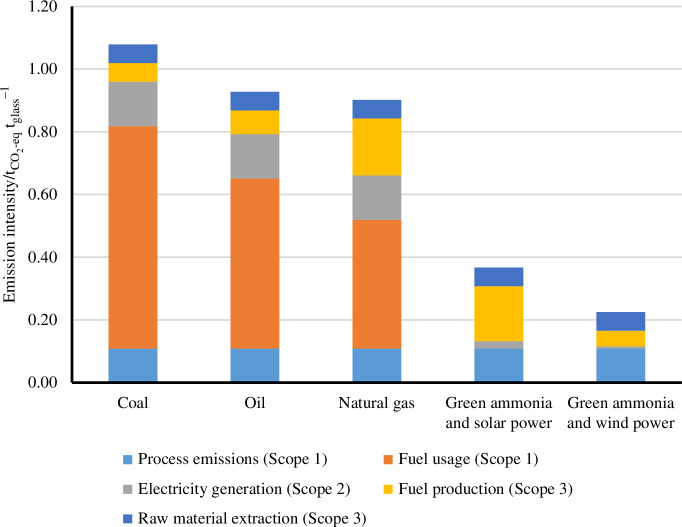
Associated CO_2_ emissions of glass making when using fossil fuels and solar- and wind-derived ammonia.

The combined replacement of fossil fuels by ammonia in melting furnaces and the replacement of current electricity production with renewable electricity has the potential of decreasing the direct CO_2_ emissions of this process between 59 and 75% (figure 8). In addition, every 10% of cullet used as inlet material can reduce the overall CO_2_ emissions by an additional 6–8%. Other relevant approaches that might be used in conjunction with ammonia as fuel are the use of pure oxygen for combustion (oxy-combustion), hybrid electrical-combustion furnaces and the use of H_2_ or H_2_/NH_3_ mixtures [31].

## 5. Cement industry

The global production of cement in 2022 was 4.16 Gt [36], making it the most consumed industrial product. Cement is used by the construction industry to form concrete when hardened after being mixed with water, sand and gravel. The production of cement is responsible for 2.3 Gt of CO_2_ per year, which accounts for ~6–8% of the global GHG emissions [37–39], and it is the main contributor to concrete emissions.

**Figure 9.**
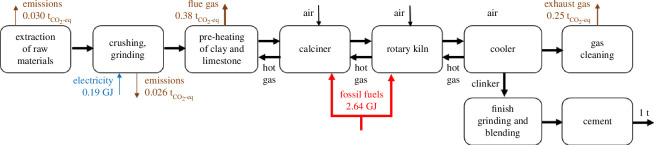
Simplified process diagram of the conventional cement production adapted from [37,40]. Key process streams are shown by black arrows. Key fuel inputs and electricity inputs are shown by red and blue arrows, respectively. CO_2_ emissions are shown by brown arrows. Values are normalized for the production of 1 ton of cement.

The main raw materials for the production of cement are clay and limestone. The process of cement production is schematically depicted in figure 9. It involves a number of physical steps where the raw materials are crushed, grinded and pre-heated before being introduced into a calciner where carbon is removed in the form of CO_2_ from limestone (CaCO_3_) to produce lime (CaO) at ~800–900°C in an endothermic process. In the next stage, the lime (CaO) reacts exothermically with SiO_2_, Al_2_O_3_ and Fe_2_O_3_ (in clay) in the kiln (up to about 1450°C) to form various silicate and oxide compounds (known as clinkers) that will make up cement. The process is heat integrated with hot gases coming from the rotary kiln used to pre-heat the incoming streams of the calciner. The net energy required in the calciner and kiln is currently provided by fossil fuels, which consist mainly of coal, heavy oils, petroleum coke and natural gas depending on the location [41].

At the moment, the process produces ~0.8 t_CO₂_/t_cement_ in an average plant, and 0.6 t_CO₂_/t_cement_ in a best-in-class one [42] where ~60% of the emissions are related to the CO_2_ liberated from the limestone in the calciner (flue gas, figure 9) and ~40% of the emissions are related to the combustion of fossil fuels as fuel (exhaust gas, figure 9).

There is a large research and innovation effort to decrease the emissions related to the use of limestone by the use of alternative raw materials such as calcium carbonate or waste products. However, a number of challenges associated to the quality, strength, long-term durability and cost of the material still remain [43–46].

### (a) Emissions reduction through the replacement of fossil fuel use with ammonia

**Figure 10.**
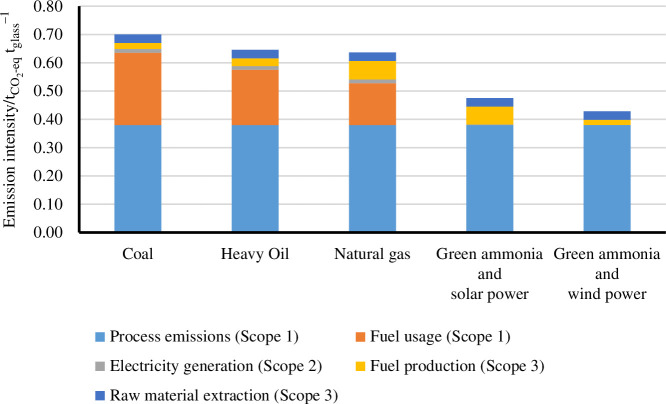
Distribution of Scopes 1, 2 and 3 of CO_2_ equivalent contributions for the cement industry considering different scenarios of fuel and electricity sources. Calculations are provided in the electronic supplementary material, S.4.

The partial replacement of fossil fuel consumption with ammonia for the production of cement has been strategically identified by Japan as a roadmap towards decarbonization [47]. Co-combustion of ammonia with coal or heavy oils has been investigated by Mitsubishi UBE Cement Corporation in collaboration with researchers from Osaka University [48,49]. Computational and experimental data suggest the feasibility of replacing up to 30% of the heavy oil mixture with ammonia without affecting the clinker composition. In addition, optimization of the fuel-oxygen ratios leads to similar NOx emissions as when using 100% heavy oil in a 10 kW furnace. Based on our calculations, the use of 30% ammonia fuel mixture in the clinker furnace leads to a 3–10% decrease in the total CO_2_ emissions/t_cement_ depending on the fossil fuel being replaced. The decrease will go to 23–55% if 100% of the fossil fuel is replaced with green ammonia. Powering the electric energy demands of the current process (used mainly for grinding and crushing) with renewable electricity can reduce emissions by 1–2% in total CO_2_ emissions/t_cement_. Results are presented in figure 10.

Electrification of the different units in the process is also currently being considered to decarbonize the production of cement, where ammonia can potentially play a role if renewable energy is used as discussed in §6. Electrification of the calciner can produce an almost pure carbon dioxide flue gas stream that can be coupled with carbon capture technology, reducing fuel-related emissions by 78% [50]. Similarly, there are opportunities to electrify the rotary kiln, which, together with an electrified calciner and carbon capture and/or utilization, can reduce CO_2_ emissions by up to 83% [51]. The first electric kiln was successfully operated by North Star Cement (Canada) before the manufacturer closed down in 2014 [52]. Currently, Europe is leading the innovation in this area with a number of companies developing relevant technologies [52]. To provide a few examples, the start-up Coolbrook (Sweden) is partnering with CEMEX on electric kilns with the first electric kiln in operation by 2024 that can reach temperatures as high as 1700°C [53]. The firm SaltX (Sweden) announced an electric arc calciner to produce green quicklime in June 2022. VTT Technical Research Center (Finland) is also developing electric rotary kilns [54]. AGICO Cement (China) has developed a kiln heated by silicon carbide heating elements [52]. As electrification is only relevant and competitive in the context of available low-cost renewable energy, the partial electrification of the process coupled with the combustion of alternative fuels is also being considered [50,51].

## 6. Green ammonia to facilitate the renewable electrification of the industry

The previous sections outlined the potential of directly using ammonia as fuel to replace the current use of fossil fuels. However, use of ammonia as fuel is limited due to its inherent properties, low flame speeds (7 cm/s at atmospheric conditions), high minimum ignition energy, narrow flammability limits and propensity to form NOx when combusted [55]. One way to circumvent these limitations is through co-firing ammonia with other fuels, making decarbonization challenging [56]. Therefore, in numerous cases, the direct electrification of different unit operations of the processes (e.g. electric kilns in cement production, electric furnaces in glass production) is being proposed as a way of decarbonization in what is so-called Power-to-X. Indeed, the chemical industry presents a number of examples such as the electrification of steam reformers (for hydrogen production) where the heat of reaction (endothermic) is provided electrically (rather than the conventional fossil fuel combustion) with an associated increase in energy efficiency [57]. Another benefit would be the replacement of steam-driven compressors by electrically driven ones that would increase their efficiency from 45 to greater than 90% with less maintenance [8,58]. Negating the use of fossil fuels in industry as energy feedstock will also impact the current heat integration strategies.

Decarbonization through electrification is only possible when renewable energy is the source of electricity. A carbon-intensive grid would result in a net increase in the CO_2_-eq emissions for process heat due to overall lower efficiencies compared with combusting fossil fuels directly. Currently, most electrical grids remain carbon-intensive, and there is a vast range of decarbonization strategies depending on the geographic availability of renewable energy. Reducing emissions by deploying solar and wind poses a significant challenge for electric grids because the energy supply is intermittent by nature. While batteries can cope with short-term variations (e.g. daily variations) they are not suitable for seasonal long-term energy storage due to their high CAPEX. Two different approaches can be envisioned to cope with the intermittent variations of renewable energy production: (i) flexibility in the energy consumption by varying the capacity of the industrial process through dynamic operation and (ii) the use of buffers to align energy supply and demand. The former case relies on the ability of the processes to ramp production capacity depending on the availability of renewable energy. In many cases, this approach requires new processes as we have demonstrated for the production of green ammonia itself [15,19]. Dynamic operation is particularly challenging in high temperature processes that rely on intricate heat integration strategies such as the production of cement, glass or steel. In this case, the use of energy buffers is likely to be mandatory. In this context, the carbon-free ammonia molecule offers advantages versus hydrogen as it is easy to store and transport, enabling the penetration of renewable energy in the industry while also promoting a trade system of renewable energy around the world that facilitates the decarbonization of regions without local renewable energy [8]. It is envisioned that green ammonia will be produced when/where renewable energy is in excess and used in power stations when renewable energy supply is insufficient to meet energy demands.

Ammonia-driven turbines in power plants are an active area of research [59]. Demonstration projects of co-firing ammonia in power plants, at a 20:80 ammonia-to-coal ratio currently exist, with Japan aiming to demonstrate 50% co-firing rates in the future and ultimately 100% ammonia power. Recently, Mitsubishi HI conducted a single-fuel burner test using an ammonia burner, and a high-ratio ammonia co-firing test with coal. In both cases, stable combustion reduced nitrogen oxide (NOx) emissions compared with coal firing and complete combustion of ammonia was achieved [60].

It is important to mention that ammonia is currently present in power plants and used as a reducing agent for the selective catalytic reduction of pollutants from combustion gases (mainly NOx) with a well-established and reliable infrastructure and technology for its storage and distribution [61,62].

The current round-trip efficiency of using ammonia to balance seasonal variations of renewable energy is ~32% (considering efficiencies of 70, 77 and 60% associated with PEM electrolyzers, Haber–Bosch ammonia production and a combined cycle gas turbine, respectively). Using solid oxide fuel cells for the conversation of ammonia into electricity will increase the overall efficiency (estimated at 39 and 50% with PEM and solid oxide fuel cells, respectively) while completely negating the production of NOx in combustion [63].

## 7. Conclusions and outlook

One of the main attractions of the ammonia molecule as a carbon-free vector of renewable energy is its flexibility in the way it can be used. With a high energy density, comparable with that of coal and other fossil fuels (see table 1), it can be directly combusted to produce heat or converted into electricity using fuel cells (e.g. PEM or SOFC) or by combustion in gas turbines in power stations.

**Table 1.** Comparison of energy density of ammonia and other fossil fuels conventionally used in industry.

fuel	energy density (MJ/kg)
ammonia	23
coal	26
heavy oil	41
natural gas	46

In this work, we have tentatively quantified the reduction of hard-to-abate industries by using green ammonia as a fuel to replace fossil fuels or as a long-term storage medium to balance the electricity grid to account for the intermittencies in production of (solar and wind) renewable energy. Considering both, the steel industry can reduce its CO_2_ emissions by between 10 and 12% when using blast furnaces and 70 and 85% when using a direct reduction and electric arc furnace when using green ammonia as fuel and electricity sources. In the case of the glass industry, the CO_2_ emissions reduction potential of green ammonia is between 59 and 75%, while in the cement industry it is between 23 and 55%. The ranges depend on the fossil fuel to be replaced as well as the source of renewable energy used for the synthesis of green ammonia, with solar-derived green ammonia having a lower CO_2_ emission reduction potential than a wind-derived one. These preliminary values of CO_2_ emission reduction potential of green ammonia in industrial processes would require further analysis, optimization and technical feasibility studies, but clearly demonstrate the appeal of this approach to continue utilizing existing high-value CAPEX assets in conventional processes. However, special care should be taken when considering the modifications of these highly integrated and optimized processes to ensure overall CO_2_ emission reductions. It is possible that in a number of cases, the replacement of fossil fuels by green ammonia can only be carried out partially in hybridized processes.

Further work should also focus on increasing the round-trip efficiency of green ammonia as energy vector with more efficient and cheaper water-splitting technologies being one of the main technological bottlenecks. From a systems approach point of view, depending on the amount of ammonia used to balance a decarbonized grid, direct combustion of ammonia can lead to higher CO_2_ emission reduction potential than direct electrification, depending on the renewable energy supply profile and demand, both inherently related to the location. In both cases, a NOx-free ammonia combustion is essential. Exciting progress in this area is the pre-combustion partial cracking of NH_3_, leading to optimum NH_3_/H_2_ mixtures with efficient combustion properties [64].

## Data Availability

Data and calculations are provided in the supplementary materials [65].
